# Single-Incision Single-Instrument Adnexal Surgery in Pediatric Patients

**DOI:** 10.1155/2015/246950

**Published:** 2015-10-07

**Authors:** Tara Loux, Gavin A. Falk, Michaela Gaffley, Stephanie Ortega, Carmen Ramos, Leopoldo Malvezzi, Colin G. Knight, Cathy Burnweit

**Affiliations:** ^1^Miami Children's Hospital, Miami, FL, USA; ^2^Herbert Wertheim College of Medicine, Florida International University, Miami, FL, USA; ^3^Florida International University, Miami, FL, USA; ^4^Miami Associates in Pediatric Surgery, Miami, FL, USA

## Abstract

*Introduction*. Pediatric surgeons often practice pediatric gynecology. The single-incision single-instrument (SISI) technique used for appendectomy is applicable in gynecologic surgery. *Methods*. We retrospectively analyzed the records of patients undergoing pelvic surgery from 2008 to 2013. SISI utilized a 12 mm transumbilical trocar and an operating endoscope. The adnexa can be detorsed intracorporeally or extracorporealized via the umbilicus for lesion removal. *Results*. We performed 271 ovarian or paraovarian surgeries in 258 patients. In 147 (54%), the initial approach was SISI; 75 cases (51%) were completed in patients aged from 1 day to 19.9 years and weighing 4.7 to 117 kg. Conversion to standard laparoscopy was due to contralateral oophoropexy, solid mass, inability to mobilize the adnexa, large mass, bleeding, adhesions, or better visualization. When SISI surgery was converted to Pfannenstiel, the principal reason was a solid mass. SISI surgery was significantly shorter than standard laparoscopy. There were no major complications and the overall cohort had an 11% minor complication rate. *Conclusion*. SISI adnexal surgery is safe, quick, inexpensive, and effective in pediatric patients. SISI was successful in over half the patients in whom it was attempted and offers a scarless result. If unsuccessful, the majority of cases can be completed with standard multiport laparoscopy.

## 1. Introduction

Laparoscopic operations for benign adnexal pathology have been conducted for many years; minimally invasive surgery was, in fact, initially pioneered by gynecologists. In most centers without specialized pediatric gynecologists, pediatric surgeons perform the majority of adnexal operations. We have previously reported our “all-in-one” single-incision single-instrument (SISI) appendectomy as an essentially scarless operation with outcomes equivalent to multiport techniques at a lower cost [1]. Since 2008, we have extended the SISI approach to adnexal pathology and found it to be a useful approach for the treatment of pediatric and adolescent ovarian or paraovarian pathology, with easy conversion to standard two- or three-port laparoscopy or even open Pfannenstiel, if additional visualization were needed.

Several authors have described the use of expensive single-site laparoscopy systems for treatment of adnexal pathology in small series of children and adolescents [2–8], and others have described similar approaches in adults [9–14], although many of these reports are for single-incision multiple-instrument methods. A few authors have also described the use of transumbilical operations for cystic ovarian lesions in small series of infants, whether laparoscopic-assisted or via minilaparotomy [15–19]. Only one other author has utilized our same approach in 15 children and adolescents [20].

A survey conducted among members of the International Pediatric Endosurgery Group (IPEG) divulged that only 7% of this highly selected group's respondents had performed single-incision pediatric endoscopic surgery (SIPES) for gynecologic pathology [21]. A quarter of respondents to this survey who did not perform SIPES at all cited inadequate resources as the reason. The purpose of the current study was to describe our high-volume, extensive experience with SISI surgery applied to adnexal pathology as a low-cost, simple, efficient, and cosmetically appealing solution for pelvic surgery in children of all ages and sizes.

## 2. Methods

Our surgical billing database was queried for Current Procedural Technology (CPT) codes indicating partial or total oophorectomy (58661, 58662, 58720, 58900, 58925, 58940) from January 1, 2008, to November 30, 2013. Five attending surgeons performed or supervised all procedures, and all surgeons had been performing SISI appendectomy for at least 2 years. We extracted data retrospectively through review of hospital and clinic charts. Data extracted included patient age and weight at time of surgery, surgical start and end times, type of surgery performed (including surgical approach at beginning, intraoperative change of approach, and reason for conversion), final pathology and cytology results, total hospital length of stay, postoperative complications, and overall length of follow-up time. This study met criteria for exempt review, determined by the Western Institutional Review Board (Case number 1-814812-1).

**Figure 1:**
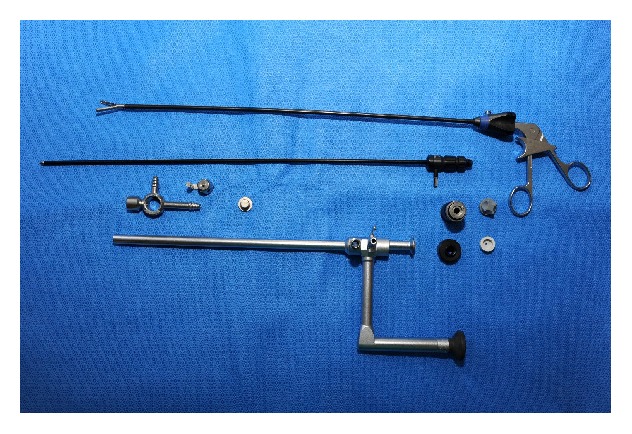
Picture of our 10 mm operating endoscope with periscope eyepiece and 5 mm instrument port.

Our SISI approach utilized a 12-millimeter standard laparoscopy trocar introduced transumbilically, after placement of an indwelling bladder catheter. We then inserted an operating endoscope (Karl Storz, product number 26036AA, Figure 1), which combines a 10-millimeter, 0-degree lens with an offset eyepiece and a 5-millimeter port through which a long instrument, such as a toothed grasper or suction, can be introduced. Ovarian torsions can almost always be reduced with this instrument. For large simple cystic lesions, aspiration of the cyst fluid was performed with a spinal needle introduced through the anterior abdominal wall under direct vision while stabilizing the lesion with the grasper or by cauterizing a hole in the cyst with the long suction and then inserting it inside the cyst. Such decompression afforded increased mobility of the adnexa. If the cystic lesion was so large that it extended out of the pelvis and could be directly visualized through the umbilical incision, it was grasped with an atraumatic clamp. A purse-string suture was then placed in the cyst wall, and a suction device was inserted for decompression of fluid. Once the adnexa was mobile, it was extruded through the umbilical incision, using a fascial extension if needed. Ovarian-sparing procedures were attempted when thought possible, with the cystic or solid lesions being dissected off the normal ovarian parenchyma, which was dropped back into the abdomen after achieving hemostasis. Total oophorectomy, sparing the fallopian tube, was utilized when normal-appearing ovarian parenchyma could not be distinguished from a solid mass of undetermined character. Rarely, full salpingooophorectomy was performed in cases of suspected malignancy. Conversion to standard laparoscopy was dependent upon the experience and comfort of the surgeon with each individual case. Alternatively, Pfannenstiel incision was performed if the surgeon deemed an open operation advisable.

Statistics utilized included the Student *t*-test, Chi-square test, and Fisher exact test where appropriate, with *p* < 0.05 considered statistically significant.

## 3. Results

We pulled 279 codes meeting the criteria from our surgical database. Eight operations had two procedure codes; five patients were excluded for nonovarian primary surgery. Four patients were found to have had a repeat operation that was not elicited in our code search. The end result was 271 evaluable operations in 258 patients, 13 of whom had recurrent adnexal pathology. The most common indications for surgical intervention were pain, cyst greater than 5 centimeters in diameter, suspected torsion, and/or solid mass. Demographic and clinical data by initial and final approach are delineated in Table 1. Over half of our initial operative attempts were via SISI (147 of 271, 54%); about half of these were converted to either standard laparoscopy or Pfannenstiel incision, resulting in 75 patients (28% of the total cohort, 51% of those started with SISI) completing treatment with the technique. The SISI method was completed in patients ranging in age from 1 day to 19.9 years (median 15.3 years) and with weights from 4.7 to 117 kilograms (median 59.4 kilograms). Reasons for change in approach are delineated in Table 2 and were dominated by desire to perform contralateral oophoropexy or presentation of a solid mass.

Twenty-two patients underwent oophoropexy (8.1% of the total cohort) at the discretion of attending surgeon, usually in the case of residual solitary ovary such as with tumor resection or from contralateral torsion with probable gonadal compromise.

With progression of time, SISI use increased and standard multiport laparoscopy use decreased; successful SISI surgery comprised only 9.3% of all operations in 2008 compared with 34% in 2013, while multiport laparoscopy decreased from 42% to 24% over the same time period. The rate of converted SISI operations was stable at around 25% each year. Successful SISI surgery was significantly shorter than all other approaches (46 mins versus 59 mins for standard laparoscopy, *t*-test, *p* < 0.001). Length of stay (0.77 days) was shorter in the successful SISI surgery group when compared with standard laparoscopy (0.92 days), but this did not reach statistical significance.

Surgical pathology results are delineated in Table 3. Only 4.7% of the cohort was found to have a malignant or premalignant condition, and these 12 cases are broken down in detail in Table 4. No case of malignant or premalignant lesion was completed with the SISI technique.

Follow-up has ranged from 0 to 72 months (median 14.2 months). Thirty minor complications occurred in 28 patients (11% of operations). SISI converted to Pfannenstiel incision had a significantly higher rate of complications (42%, *p* < 0.002) than either successful SISI (6.7%), SISI converted to multiport laparoscopy (15%), initial multiport laparoscopy (8.2%), or initial Pfannenstiel approach (6.3%). There were no major complications of surgery, although there were five recurrences of benign disease scattered across four of the different surgical techniques, which were all treated with repeat operation. Two deaths occurred in our cohort during long-term follow-up, one due to overwhelming oncologic disease (Burkitt lymphoma) and the second from peritonitis 7 months postoperatively. This was associated with a peritoneal dialysis catheter and unrelated to the ovarian pathology.

In regard to cytologic collection from peritoneal washings or cyst aspiration, 83 of 271 patients in the cohort (31%) had samples generated. In all but one case, the specimen yielded benign or nondiagnostic results. Cytology was sent in only 6 of 12 malignant or premalignant lesions, and a single specimen in a girl with a yolk sac tumor showed atypical cells.

## 4. Discussion

We report here our series of single-incision, single-instrument (SISI) operations for adnexal pathology in infants, children, and adolescent girls. Our series is the largest reported in the English-language literature to date. We feel our approach is useful as a cost-effective, simple, safe, and cosmetically appealing procedure for treating gynecologic pathology in children. We have gone on to utilize our SISI approach for other pathologies as well, including resection of Meckel diverticulum and duplications, reduction of intussusception, segmental colon resection, treatment of empyema, and extraction of multiple gastric or intestinal tract magnets. We feel this technique is broadly applicable and are continuously looking for new ways to safely limit expenses and improve outcomes with its employment.

One of the major decisions both prior to initiating a SISI approach and once inspecting the pelvic pathology is whether or not there is an immature or cancerous component to the pathology, for avoidance of rupture in these cases may be critical. We have found that the majority of cases can be approached with SISI, as long as the radiology is consistent with benign pathology (i.e., simple appearing cyst without solid component, hemorrhagic cyst, torsion, or solid mass consistent with teratoma based on appearance of fat or calcifications) and the hormonal markers are normal. Any indication of malignancy (peritoneal studding, retroperitoneal lymphadenopathy, noncalcified or highly vascularized solid mass, or elevated hormonal markers) should discourage use of the SISI technique. In these situations, multiport or multi-instrument single-incision laparoscopy (e.g., LESS) can be considered, although the standard is open surgery.

Intraoperatively, additional care must be taken as risk of spill in ovarian malignancy must be avoided with aspiration or attempts at mobilization. Intracorporeal needle decompression or periumbilical mobilization with purse-string evacuation is only undertaken in simple cystic lesions without solid component, unless the lesions are small and mobile enough to extrude easily through the umbilicus. In the present series, it is noteworthy that while malignancy was uncommon, no patient with malignancy was completed with the SISI technique. In cases of suspected malignancy or uncertain pathology, multiport laparoscopy with endobag extraction or Pfannenstiel incision is utilized. This latter option affords the opportunity to extracorporeally attempt the tedious dissection often required to perform an ovarian-sparing procedure while guarding against intra-abdominal rupture in benign but very large tumors such as teratomas. Occasionally, with a very large or clearly malignant tumor, we utilized a midline laparotomy.

Teratomas form an interesting group of neoplasms for which the SISI technique is particularly applicable. Usually mature but occasionally massive, they may be bilateral in 10% of cases [22], whether synchronously or metachronously. We follow these patients for a period of at least 5 years after diagnosis, which allows small, metachronous lesions to be identified early, making ovarian preservation easier. Small ovarian dermoids found during surveillance are easily treated using SISI instrumentation to exteriorize the ovary through the umbilicus for excision.

Based on the data gleaned from our sizable cohort, and contrary to the accepted practice in adult patients, peritoneal washing and/or cyst fluid collection for cytology may be unnecessary when benign disease is suspected. In no case did the results change either the diagnosis or the treatment, while adding a significant cost to the patient's care. Larger studies would be needed before recommending the abandonment of this diagnostic modality, but a prospective, multicenter investigation of cytology in pediatric adnexal pathology would be a fertile area for future investigation.

Limitations of our study include its retrospective and therefore descriptive nature. The conclusions herein are meant to encourage the application of this technique, while recognizing that surgeon judgment will always be principal when considering operative approach. Our report, the largest reported experience to date, reveals that a significant subset of girls with adnexal pathology can undergo successful treatment with the single-incision single-instrument technique, allowing for scarless surgery, minimal discomfort, and same-day discharge. We advocate further implementation of this safe, low-cost, and cosmetically appealing solution to the treatment of pediatric surgical illness.

## Figures and Tables

**Table 1:** Select demographic characteristics of patients with ovarian pathology undergoing operation.

Operative technique (initial approach : final approach)						
Demographic variable	SISI : SISI	SISI : multiport	SISI : Pfannenstiel	Multiport : multiport	Multiport : Pfannenstiel	Open
Number of pt. (% of cohort)	75 (27.7)	53 (19.6)	19 (7.0)	97 (35.8)	5 (1.8)	22 (8.1)
Mean age, yrs (range)	14.6 (1 d–19.9 y)	14.2 (1 m–9.6 y)	14.9 (9.7–18.8 y)	14.5 (3 m–9.9 y)	12.7 (8.3–15.1 y)	13.3 (3.8–18.2 y)
Mean weight, kgs (range)	61.0 (4.7–117)	62.7 (4.6–110)	65.3 (30.5–117)	63.5 (5.6–140)	64.1 (28.6–106)	53.8 (19.2–93)

**Table 2:** Reason for change in approach, by initial approach.

Reasons for change	Number of patients		
	SISI : multiport	SISI : Pfannenstiel	Multiport : Pfannenstiel
Oophoropexy	19		
Solid mass	7	16	4
Inability to mobilize adnexa	6		
Large size	4	2	1
Additional procedure	4		
Bleeding	3		
Adhesions	1	1	
Better visualization	3		
No reason given	6		
Total	**53**	**19**	**5**

**Table 3:** Surgical pathology and cytology results.

Pathology	Number of patients (*n* = 255)^*∗*^	Percentage of cohort	Cytology	Number of patients (*n* = 263)^*∗∗*^	Percent of cohort
Simple cyst or serous cystadenoma	84	32.9%	No specimen sent	188	71.5%
Mucinous cystadenoma	9	3.5%	Benign mesothelial, epithelial, mucin-producing, or ciliated tubal cells	41	15.6%
Hemorrhagic follicle	37	14.5%	Degenerated cells	6	2.3%
Ovarian torsion	17	6.7%	Various benign leukocytes	6	2.3%
Normal ovary	2	0.8%	Benign cystic teratoma	1	0.4%
Paratubal or paraovarian cyst	35	13.7%	Mesothelial cells and leukocytes	3	1.1%
Hydrosalpinx	4	1.6%	Red blood cells	10	3.8%
Mature teratoma	55	21.6%	Nondiagnostic (no cells)	7	2.7%
Malignant or premalignant lesion	12	4.7%	Atypical cells	1	0.4%

^*∗*^For 1 patient no report was available and in 11 operations no specimen was sent.

^*∗∗*^In 8 patients no report was available.

**Table 4:** Breakdown of clinical and pathology characteristics of patients with malignant or premalignant lesions (4.7% of total patients).

Patient number	Age (yrs)	Weight (kg)	Timing of operation	Initial operative approach	Final operative approach	Reason for change	OR time (hr:min)	Side of pathology	Reason for operation	Final surgical pathology
3	17.8	105	Urgent	SISI	Multiport laparoscopy	Solid mass	1:51	Right	Hirsutism, solid mass	Sex cord stromal tumor
57	15.5	88.2	Elective	SISI	Multiport laparoscopy	Unable to mobilize	1:31	Right	Cyst > 5 cm	Low grade mucinous tumor of ovary
66	13.9	50	Urgent	Multiport laparoscopy	Multiport laparoscopy	No change	2:10	Left	Pain, solid mass	Yolk sac tumor
99	17.2	48	Urgent	Midline	Midline	No change	1:38	Right	Bowel obstruction, solid mass	Burkitt lymphoma
117	16.7	61	Urgent	Midline	Midline	No change	1:47	Right	Bowel obstruction, solid mass	Dysgerminoma
126	3.8	19.2	Urgent	Midline	Midline	No change	1:05	Right	Pain, solid mass	Sex cord stromal tumor
144	15.2	93	Elective	Midline	Midline	No change	2:15	Right	Ascites, pleural effusions, solid mass	Sex cord stromal tumor
163	14.2	38.7	Elective	Multiport laparoscopy	Multiport laparoscopy	No change	0:55	Bilateral	Turner syndrome, virilization	Dysgenetic gonads
198	11.9	33.5	Urgent	Pfannenstiel	Pfannenstiel	No change	0:33	Left	Cyst > 5 cm, solid mass	Immature teratoma
215	2.9	16.8	Elective	Multiport laparoscopy	Multiport laparoscopy	No change	1:03	Right	Precocious puberty, solid mass	Steroid-producing ovarian tumor
220	14.4	53.6	Urgent	Pfannenstiel	Pfannenstiel	No change	1:08	Left	Cyst > 5 cm	Borderline cystic mucinous tumor
270	8.6	32	Elective	Pfannenstiel	Pfannenstiel	No change	0:59	Left	Solid mass	Immature teratoma
